# Opposite effects of angiotensins receptors type 2 and type 4 on streptozotocin induced diabetes vascular alterations in mice

**DOI:** 10.1186/1475-2840-13-40

**Published:** 2014-02-10

**Authors:** Mohamad Nasser, Nicolas Clere, Laurent Botelle, James Javellaud, Nicole Oudart, Sébastien Faure, Jean-Michel Achard

**Affiliations:** 1 INSERM, UMR-S850, Université de Limoges, 2 rue du Docteur Marcland, 87025 Limoges Cedex, France; 2 INSERM UMR 1063, Université d’Angers, IRIS, Rue des Capucins, Angers, France; 3 Laboratoire de Physiologie, Faculté de Médecine, 2 rue du Dr Marcland, 87000 Limoges, France

**Keywords:** Angiotensin IV, AT2 receptor, AT4 receptor, Streptozotocin-induced diabetes, Endothelial dysfunction

## Abstract

**Background:**

We examined the effect of chronic administration of angiotensin IV (AngIV) on the vascular alterations induced by type 1 diabetes in mice.

**Methods:**

Diabetes was induced in adult Swiss mice with a single injection of streptozotocin (STZ). Mice were treated subcutaneously with AngIV (1.4 mg/kg/day) either immediately following diabetes induction (preventive treatment), or treated with AngIV (0.01 to 1.4 mg/kg), alone or with the AT4 receptor antagonist Divalinal or the AT2 receptor antagonist PD123319, for two weeks after 4 weeks of diabetes duration (rescue treatment). Acetylcholine-induced, endothelium-dependent relaxation (EDR) was measured in isolated aortic rings preparations. Histomorphometric measurements of the media thickness were obtained, and nitric oxide (NO) and superoxide anion production were measured by electron paramagnetic resonance in aorta and mesenteric arteries. The effect of diabetes on mesenteric vascular alterations was also examined in genetically modified mice lacking the AT2 receptor.

**Results:**

Induction of diabetes with STZ was associated with a progressive decrease of EDR and an increase of the aortic and mesenteric media thickness already significant after 4 weeks and peaking at week 6. Immediate treatment with AngIV fully prevented the diabetes-induced endothelial dysfunction. Rescue treatment with AngIV implemented after 4 weeks of diabetes dose-dependently restored a normal endothelial function at week 6. AngIV blunted the thickening of the aortic and mesenteric media, and reversed the diabetes-induced changes in NO and O_2_^•–^ production by the vessels. The protective effect of AngIV on endothelial function was completely blunted by cotreatment with Divalinal, but not with PD123319. In contrast, both the pharmacological blockade and genetic deletion of the AT2 receptor reversed the diabetes-induced morphologic and endothelial alteration caused by diabetes.

**Conclusions:**

The results suggest an opposite contribution of AT2 and AT4 receptors to the vascular alterations caused by streptozotocin-induced diabetes in mice, since chronic stimulation of AT4 by AngIV and inhibition of AT2 similarly reverse diabetes-induced endothelial dysfunction and hypertrophic remodeling, and increase NO bioavailability.

## Background

Diabetes mellitus is a major cause of morbidity and mortality worldwide and is associated with an increased risk of cardiovascular disease [1]. Indeed, cardiovascular disorders in diabetic patients include premature atherosclerosis, manifest as myocardial infarction and stroke as well as impaired cardiac function, predominantly diastolic dysfunction [2]. The precise initiating event of these disorders is unknown although dysfunction within the endothelium is thought to be an important early contributor. The endothelium is crucial for maintenance of vascular homeostasis, ensuring that a balance remains between vasoactive factors (such as angiotensin II (AngII) and nitric oxide (NO)), controlling its permeability, adhesiveness, and integrity, but this balance appears compromised by diabetes [3,4].

AngII, the main bioactive peptide of the renin angiotensin system (RAS), plays a pivotal physiological role in the regulation of salt homeostasis, kidney function and blood pressure through binding to AT1 receptor. It has been discovered that besides its systemic regulatory functions, AngII subserves paracrine and autocrine actions in various organ and tissues, including the heart and the vessels, where it is locally released [5]. AngII was shown to stimulate the expression of leukocytes adhesion molecule, and the release of inflammatory cytokines. Moreover, AngII increases the oxidative stress thereby impairing the physiological endothelial balance between NO and reactive oxygen species (ROS). AngII thus emerged as a pivotal mediator of pathophysiological mechanisms involving functional endothelial impairment and vascular hypertrophy in a number of clinical disorders such as essential hypertension [6], atherosclerosis [7], and diabetes [8].

Reducing AngII formation or signaling with angiotensin converting enzyme inhibitors (ACEIs) or AT1 receptor antagonists (ARBs) thus appeared as an ideal therapeutic approach, expected to provide beneficial protective effect beyond the blood pressure reduction. Although both class of drugs have been proven effective to lower cardiovascular morbidity and mortality in large clinical trials [9,10], further therapeutic improvements still remain needed to reduce the excessive burden of cardiovascular diseases, especially in diabetes patients.

During the last decade, it has become increasingly clear that the renin-angiotensin-aldosterone system (RAAS) does not resume to AngII and the AT1 receptor, and that other receptors and angiotensin peptides modulate and mitigate or counteract the action of AngII. Studies conducted in two AT2-null mice strains [11,12] suggested that the alternative receptor to AngII, AT2, counter regulates the AT1-mediated antinatriuretic and pressor actions of AngII, by a mechanisms involving increased NO production. This conclusion was further supported by pharmacological studies in rat [13-15]. Furthermore, other angiotensin peptides, such as angiotensin (1-7) and angiotensin (3-8) (AngIV), acting through their respective specific receptors, *mas* and AT4, also elicit effects that oppose the AngII AT1-mediated action. AngIV increases blood flow in the renal cortex and the brain, involving a vasodilator effect. The AngIV-induced vasodilation is dependent on nitric oxide release, since pretreatment with L-NAME abolished the increase of cerebral and renal cortical blood flow [16,17].

An integrative understanding of the complex interactions within the RAAS would certainly be helpful to better identify therapeutic targets and refine RAS pharmacological modulation in order to improve cardiovascular prevention. Of importance, the fact that AngIV acutely administered opposes the systemic antinatriuretic and pressor effect of AngII does however not necessarily imply that it could balance the chronic deleterious vascular effects of AngII at the tissue level. An important pioneer step forward was thus made recently with the report by Vinh et al. that chronic AngIV treatment reverses endothelial dysfunction in ApoE-deficient mice fed a high fat diet [18]. The same group further evidenced that in this experimental model of atherosclerosis, AngIV treatment was able to restore endothelial function even when administered in mice with advanced atheroma [19]. Then, the objective of the present study was to examine if AngIV plays a similar protective role against endothelial dysfunction and vascular hypertrophy in the mice model of type 1 diabetes induced by streptozotocin.

## Methods

### Animals

Swiss Male mice (20–30 g) were obtained from Depre (Saint Doulchard, France). Additional experiments were conducted in FVB/N mice (Institut für Experimentelle und Klinische Pharmakologie und Toxikologie, Freiburg, Germany) with deleted AT2R (Agtr2^-/-^) gene that were compared to Agtr2^+/+^ mice [11]. Mice were maintained in a room under controlled temperature (23°C), humidity and air flow conditions, with a fixed 12-h light-dark cycle, food and water available *ad libitum*. The current investigation conformed to the guidelines for ethical care of experimental animals of the European Community and was approved by the French Agriculture Ministry (authorization number B-00889).

### Experimental design

Diabetes was induced by a single intraperitoneal injection of streptozotocin (STZ; 200 mg/kg) in citrate buffer (pH 4.5) during the fasting state. Control animals received equivalent doses of the citrate buffer solution. Hyperglycemia occurred 2 days after STZ injection and was verified using an Accu-Check Active glucometer (Roche, Lyon, France). Mice were considered to be diabetic and were included in the experimental diabetic groups when blood glucose was ≥300 mg/dL (i.e. 16,6 mmol/L) 48 hours after the STZ injection. To examine the time course of diabetes-induced vascular alterations, three groups of diabetic mice were sacrificed after respectively 4, 6 and 8 weeks of diabetes duration.

### Treatments

#### *Preventive treatment with AngIV*

Mice were anaesthetized with isoflurane 5% and an osmotic mini-pumps (Alzet® model 2004, Palo Alto, CA) containing sterile saline vehicle (NaCl 150 mmol/L) or AngIV (1.4 mg/kg/day) were implanted subcutaneously in the back of the neck for 4 weeks. Induction of diabetes with streptozotocin was performed immediately after the surgical procedure.

#### *Rescue treatment with AngIV*

Other groups of diabetic mice were left untreated for 4 weeks after diabetes induction, and the various treatments were implemented for two additional weeks. In these mice with already established diabetes, the surgical implantation of an osmotic pump was not possible and the drugs were administered by a daily subcutaneous injection. Separate groups of mice were treated with AngIV (0.01; 0.03; 0.1; 0.3; 0.7 and 1.4 mg/kg/d), whereas control diabetic group received daily injection of the vehicle (NaCl 150 mmol/L). In parallel, two groups of diabetic mice treated with AngIV (1.4 mg/kg/d) were cotreated with the specific AT4 receptor antagonist Divalinal (2 mg/kg/d) or the AT2 specific antagonist PD123319 (20 mg/kg/d) *via* subcutaneous injection. Finally, a set of experiments was conducted in Agtr2^+/+^ or Agtr2^-/-^ with or without STZ-induced diabetes.

### Analytical methods

Weight and blood glucose of each mouse were recorded before the sacrifice. Systolic blood pressure (SBP) was measured at the end of treatment using non-invasive tail-cuff apparatus (Bionics Instrument CO., LTD) in anaesthetized mice. SBP was averaged from three consecutive measurements taken at intervals of 2-3 min.

### Measurement of vascular reactivity of aortic rings and mesenteric arteries

After sacrifice of mice, the thoracic aorta and the mesenteric artery were removed and placed in Krebs Bicarbonate Buffer (KBF, PH 7.4) consisting of (mM): NaCl 118, KCl 3.7, KH_2_PO_4_ 1, MgCl_2_ 1.2, CaCl_2_ 1.4, NaHCO_3_ 25, and glucose 11). The arteries were cleaned from fat and connective tissues. First, aortic rings (3 mm in length) were mounted isometrically, each ring was suspended between two 100 μm stainless steel wires connected to an isometric force transducer (Powerlab, AD Instruments). Aortic preparations were placed in chambers, kept at 37°C and bubbled continuously with 95% O2 – 5% CO_2_ in Krebs Bicarbonate Buffer (KBF) (pH 7.4). Tissues were stretched to their optimum tension of 0.75 g and equilibrated and then washed with fresh KBF at 15 min intervals for 60 min. To determine endothelium–dependent relaxation, vessels were maximally pre-contracted with phenylephrine (Phe; 10^-6^ M) before cumulative applications of acetylcholine (ACh; 10^-9^ to 3.10^-5^ M). Finally, the vessels were subjected to 10^-5^ M sodium nitroprusside (SNP), a donor of NO.

Second, mesenteric artery segments were dissected and mounted on a wire-myograph (DMT, Aarhus, DK) as previously described [20]. Briefly, two tungsten wires (40 μm diameter) were carefully inserted into the arterial lumen, and fixed to a force transducer and a micrometer, respectively. Arteries were bathed in the PSS as described above, and set to the baseline circumference. After stabilizing for 45 min, artery viability was tested using a potassium-rich solution (80 mM). Acetylcholine (ACh) cumulative concentration–response curves (10^-9^ to 10^-5^ M) were then obtained after phenylephrine-induced preconstriction (50% of maximal contraction).

### Histomorphometric analysis

Sections of thoracic aorta and mesenteric artery were incubated at 37°C in the physiological saline solution calcium free (composition in mM: NaCl 130; KCl 3.7; KH_2_PO_4_ 1.2; NaHCO_3_ 14.9; MgSO_4_ 1.2; EGTA 2; glucose 11) and supplemented with papaverin (10^-6^ M) and SNP (10^-5^ M). After 20 minutes of incubation, these arteries were fixed in 4% formaldehyde for 30 minutes. Transverse sections were immersed in Tissue-Tek (Sakura, Alphen aan den Rijn, The Netherlands) and snap frozen in liquid nitrogen, than stored at -80°C until they were cut into 7 μm thick. Sections were placed on Superfrost® slides and were stained with orcein to view internal and external elastic lamina. An image capture (200× magnification) was performed on these sections using an optical microscope (Olympus IMT-2) with a camera (Color Video Camera, Sony). The thickness at 10 different points of the wall media was measured and the media thickness was calculated, as previously described [21].

### NO and superoxide anion (O_2_• –) determinations by electron paramagnetic resonance

Detection of NO production was performed using Fe^2+^ diethyldithiocarbamate (DETC; Sigma–Aldrich) as spin trap [22]. Briefly, arteries were treated with 250 μL of colloid Fe (DETC)_2_ and incubated for 45 min at 37°C. Arteries were then frozen in plastic tubes. NO detection was measured *in situ* by electron paramagnetic resonance (EPR). Values are expressed as amplitude of signal per weight. For O_2_^•–^ quantification, arteries were allowed to equilibrate in deferoxamine-chelated Krebs–acide 4-(2-hydroxyéthyl)-1-pipérazine ethane sulfonique solution containing 1-hydroxy-3-methoxycarbonyl-2,2,5,5-tetramethylpyrrolidin (Noxygen, Mainz, Germany) (500 μmol/l), deferoxamine (25 μmol/l) and DETC (5 μmol/l) under constant temperature (37°C) for 20 min. Arteries were then frozen in plastic tubes and analyzed by EPR spectroscopy. Values are expressed as units per mg of artery. Sections of thoracic aorta and mesenteric artery were subjected to electron spin resonance (ESRR) spectroscopy (Magnettech miniscope MS200, Germany) to quantify O_2_^•–^ and NO production.

### Drugs

Phenylephrine, acetylcholine, streptozotocin, sodium nitroprusside (SNP), indomethacin, NG-nitro-L-arginine methyl ester (L-NAME), DETC and deferoxamine were purchased from Sigma-Aldrich (France). CMH was purchased from Noxygen (Germany) and PD123319 was purchased from Tocris Bioscience (United Kingdom). Divalinal was a kind gift from Dr Jay Wright (Washington State University, USA). Stock solutions of phenylephrine, acetylcholine, SNP, and L-NAME were dissolved in distilled water, streptozotocin was dissolved in citrate buffer and PD123319 and Divalinal were dissolved in water for injection. CMH, DETC and deferoxamine were dissolved in Krebs-buffer deoxygenated by nitrogen flow.

### Data analysis

Results were expressed as mean ± SEM. Relaxation of arteries was expressed as percentage of maximal relaxation induced by sodium nitroprusside (10^-5^ M). For O_2_^•–^ and NO detections, results were expressed in mean of the ratio in absorbance over the dry weight in mg (A/mg dry weight). The concentration-response curves were assessed by analysis of variance (ANOVA) with repeated measures, followed by Bonferroni post-hoc tests. Other data were analyzed by analysis of variance (ANOVA). P < 0.05 indicates significant difference.

## Results

### Metabolic and vascular alterations induced by streptozotocin-induced diabetes

**Figure 1:**
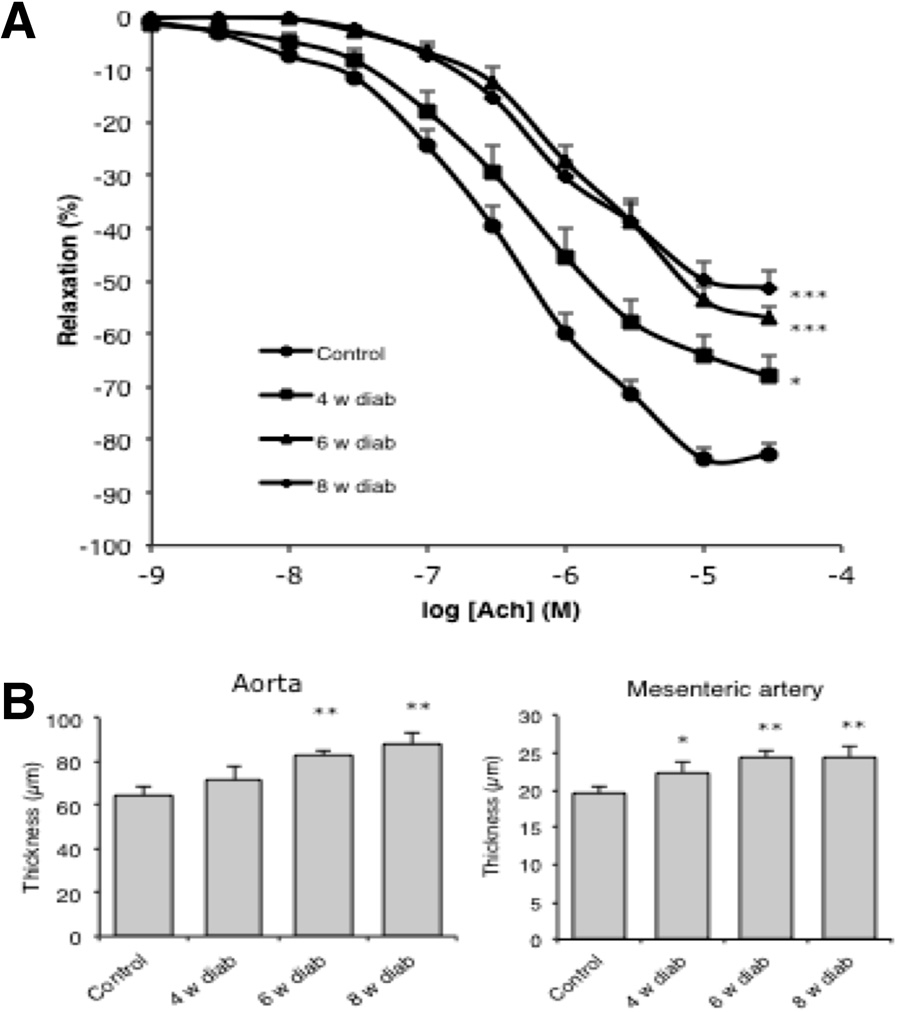
**Vascular alterations induced by diabetes.** Time course of the reduction of endothelium-dependent relaxations in the aorta after 4, 6 and 8 weeks of diabetes **(A)**. Time course of the increase of aortic and mesenteric media thickness **(B)**. *p < 0.05; **p < 0.01; ***p < 0.001 *versus* control.

Injection of streptozotocin caused a threefold elevation of blood glucose by comparison with the control mice at four, six and eight weeks of diabetes. Furthermore, streptozotocin-induced diabetes caused a progressive loss of weight from 4 to 8 weeks and a significant decrease of systolic blood pressure (Table 1). After 4 weeks of diabetes, aortic rings of the diabetic mice showed a significant blunting of Acetylcholine-mediated endothelium-dependent relaxation (EDR). EDR further impaired progressively with diabetes duration, peaking at 6 weeks (Figure 1A). In contrast, the SNP-induced endothelium-independent relaxations were not different between control and diabetic aortas, even after 8 weeks of diabetes (*data not shown*). Morphologic alterations paralleled the endothelial dysfunction, with a progressive significant increase of thoracic aorta and mesenteric artery media thickness, in comparison with control vessels (Figure 1B).

**Table 1:** Body weight, blood glucose and systolic blood pressure

**Groups**	**Body weight**	**Blood glucose**	**SBP**
**1st study**			
Control	38 ± 1*	8.8 ± 0.3*	85 ± 3*
4 w diabetes	28 ± 1	30.9 ± 0.7	68 ± 5
4 w diabetes - angIV	29 ± 1	30.6 ± 0.4	66 ± 3
6 w diabetes	29 ± 1	29.7 ± 0.8	67 ± 3
6 w diabetes - angIV	26 ± 1	30.4 ± 1.1	69 ± 6
8 w diabetes	25 ± 1	33.4 ± 0.8	69 ± 4
**2nd study**			
Agtr2+/+	32 ± 1 #	9.8 ± 0.8 #	87 ± 3 #
Agtr2+/+ diabetes	25 ± 1	29.3 ± 1.2	67 ± 5
Agtr2-/-	31 ± 1.4 #	9.7 ± 0.6 #	88 ± 3 #
Agtr2-/- diabetes	25 ± 1.4	29.6 ±1.3	66 ± 5

Body weight (g), blood glucose (mmol/l) and systolic blood pressure (mmHg) are expressed as the mean ± S.E.M, (n = 8-10 per group). Statistical significance indicated by *p < 0.05 vs. control; # p < 0.05 vs diabetes.

### Angiotensin IV prevents diabetes-induced endothelial dysfunction

**Figure 2:**
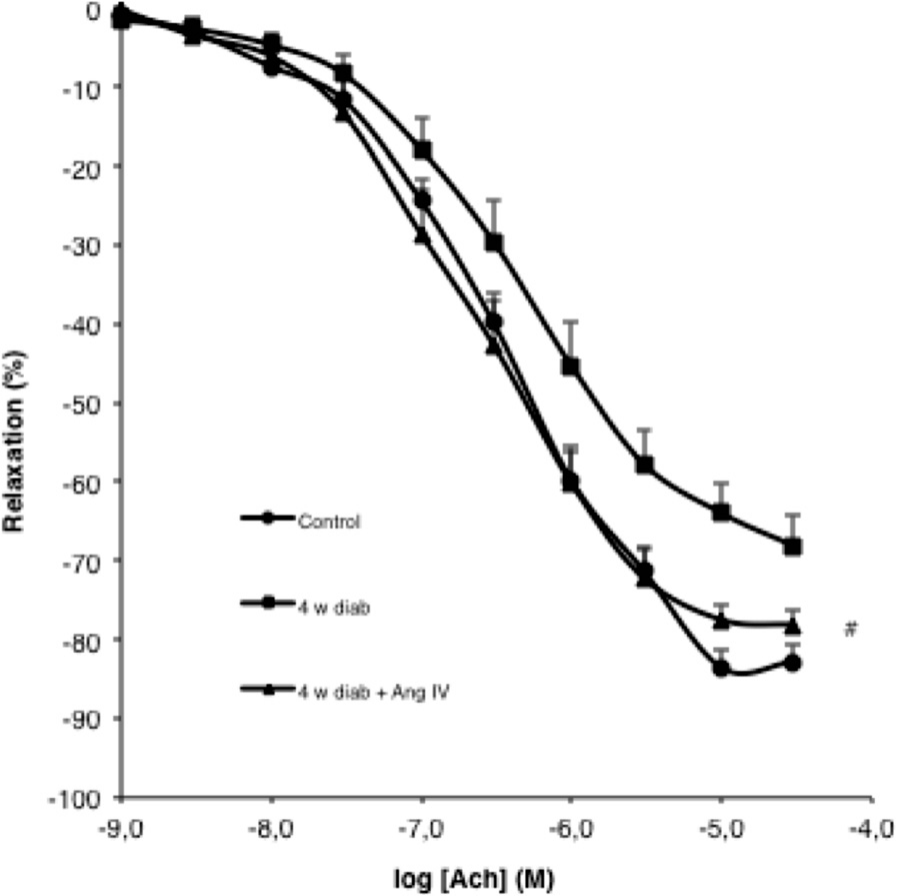
**Chronic AngIV treatment prevents endothelial dysfunction.** Concentration-response curves for ACh in aorta for control, 4 weeks diabetes, and 4 weeks diabetes treated with AngIV (1.4 mg/kg/d). Mice were treated at the beginning of diabetes for a period of 4 weeks**.** Statistical significance between 4 weeks diab and 4 weeks diab + AngIV indicated by # p < 0.05.

Mice were treated with subcutaneous administration of AngIV (1.4 mg/kg/d) *via* an osmotic pump implanted in the back of the neck immediately prior to induction of diabetes for 4 weeks. AngIV had no effect on blood glucose levels, weight loss and blood pressure (Table 1). However, at week 4, the decrease of endothelium dependent relaxation induced by diabetes was completely prevented by AngIV (Figure 2).

### Angiotensin IV reverses diabetes-induced endothelial dysfunction

**Figure 3:**
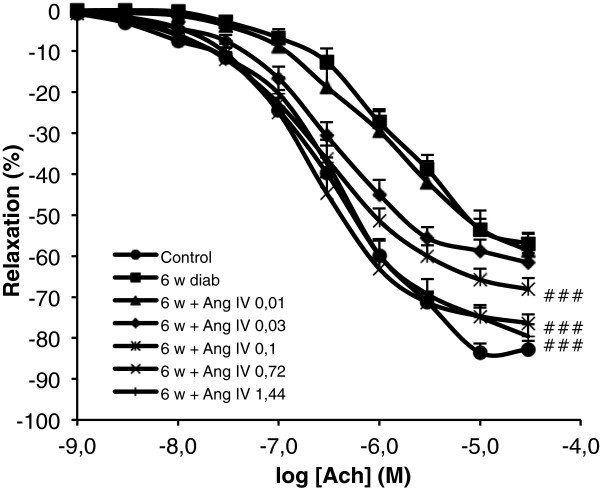
**Dose dependent effect of chronic treatment with AngIV in 6 weeks diabetic mice on endothelial function.** Concentration-response curves for ACh in thoracic aorta for control, 6 weeks diabetic mice, and 6 weeks diabetic mice treated during the last two weeks with AngIV (0.01 or 0.03 or 0.1 or 0.7 or 1.4 mg/kg/d). Statistical significance between 6 weeks diab and 6 weeks treated groups indicated by # # # p < 0.001.

To determine if AngIV, beyond its preventive effect, could reverse the vascular alterations induced by diabetes, the effect of a rescue treatment implemented after 4 weeks of diabetes has been examined. AngIV was administrated by a single daily subcutaneous injection of AngIV (1.4 mg/kg/d in saline) for 15 days, whereas control mice were treated with the vehicle. Since the treatment was effective and fully restored aortic endothelium-dependent relaxation at the level of the non-diabetic controls, additional groups of mice were treated with decreasing doses of AngIV to establish the dose-response curve. As shown in Figure 3, a significant shift to the left of the Ach-induced relaxation was already observed at the dose of 0.03 mg/kg, whereas 0.7 mg/kg was sufficient to fully restore EDR at the level of non-diabetic controls.

### Role of AT2 and AT4 receptors

**Figure 4:**
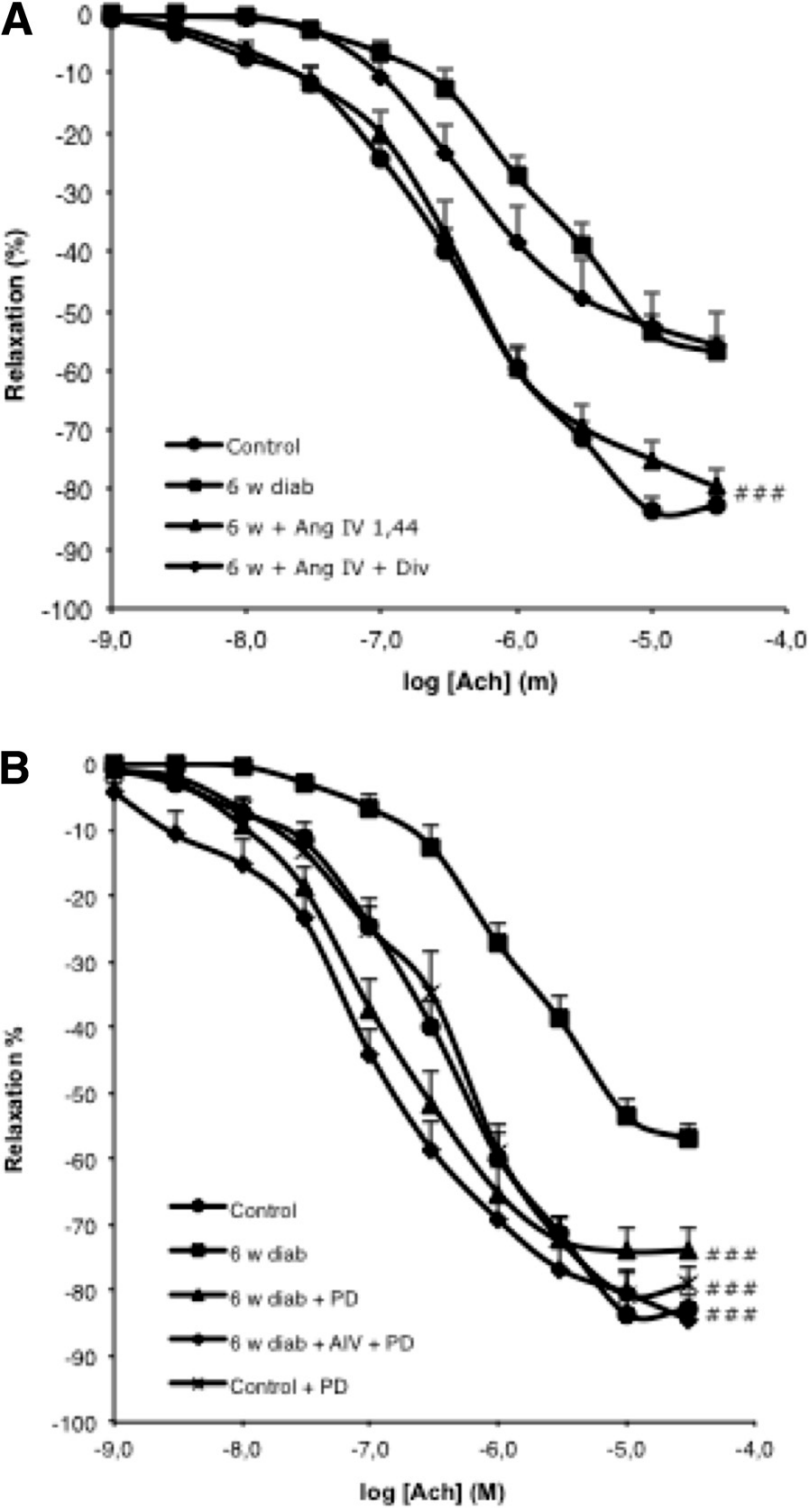
**Effect of chronic cotreatment with AngIV or PD123319 in 6 weeks diabetic mice on endothelial function. (A)** Mice were treated for two weeks after 4 weeks of diabetes initiation with vehicle (6 w diab), AngIV 1.4 mg/kg/day alone (6 w diab + AngIV), or combined with the specific AT4 antagonist Divalinal 2 mg/kg/d (6 w diab + AngIV + Div). # # # p < 0.001 for AngIV + Divalinal versus AngIV alone. **(B)** Mice were treated for two weeks after 4 weeks of diabetes initiation with the specific AT2 antagonist PD123319 20 mg/kg/d (6 w diab + PD) alone or in combination with AngIV (6 w diab + AngIV + PD). #*versus* (diab).

To determine the respective role of AT2 and AT4 receptors, two groups of 4 weeks diabetes mice were co-treated with AngIV (1.4 mg/kg/d) and respectively the AT4 receptor antagonist Divalinal (2 mg/kg/d) or the specific AT2 receptor antagonist PD123319 (20 mg/kg/d) for 15 days. Divalinal completely blunted the protective effect of AngIV on aortic endothelium-dependent relaxation (Figure 4A). In contrast, treatment with PD123319 did not abrogate the protective effect of (Figure 4B). Since these results were unexpected, we examined the effect of PD123319 on EDR in diabetic and control mice in the absence of AngIV. PD123319 had no effect on EDR in control mice (*not shown*), but completely reversed the decrease in EDR induced by diabetes (Figure 4B).

### Effect of AngIV and AT2 receptor blockade on diabetes-induced vascular remodeling

**Figure 5:**
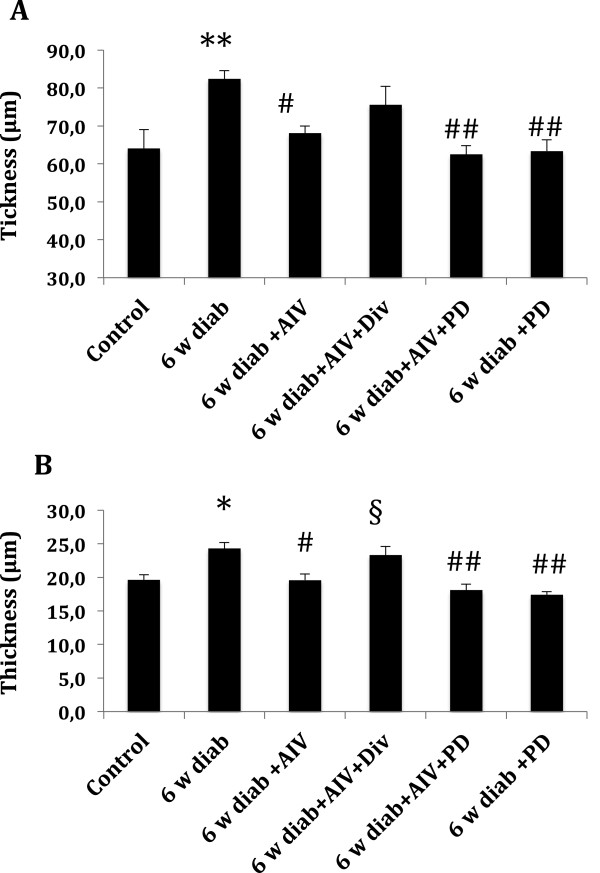
**Tunica media thickness.** Sections for thoracic aorta **(A)** and mesenteric artery **(B)** for control group (control) and 6 weeks diabetes groups treated for the last two weeks with vehicle (6 w diab), AngIV (6 w diab + AIV), AngIV and Divalinal (6 w diab + AIV + Div), AngIV and PD123319 (6 w diab + AIV + PD), or PD123319 alone (6 W diab + PD). *p <0.05 and **p < 0.01 *versus* control; # p < 0.05 and # # p < 0.01 *versus* 6w diab, § p < 0.05 *versus* 6w diab + AIV.

Analysis of the sections of thoracic aorta (Figure 5A) and mesenteric arteries (Figure 5B) showed that in 6 weeks diabetes mice, treatment with AngIV prevented the increase of the media thickness. Also, blockade of the AT2 receptor with PD123319 from weeks 4 to 6 was equally effective as AngIV to prevent the increase of aortic and mesenteric media thickness induced by diabetes. The protective effect of AngIV on media thickening was abolished in mesenteric arteries, and was non-significantly attenuated in the aorta sections. In contrast, in both vascular beds, the protective action of AngIV was unaffected by cotreatment with PD123319.

### Production of nitric oxide and superoxide anion

**Figure 6:**
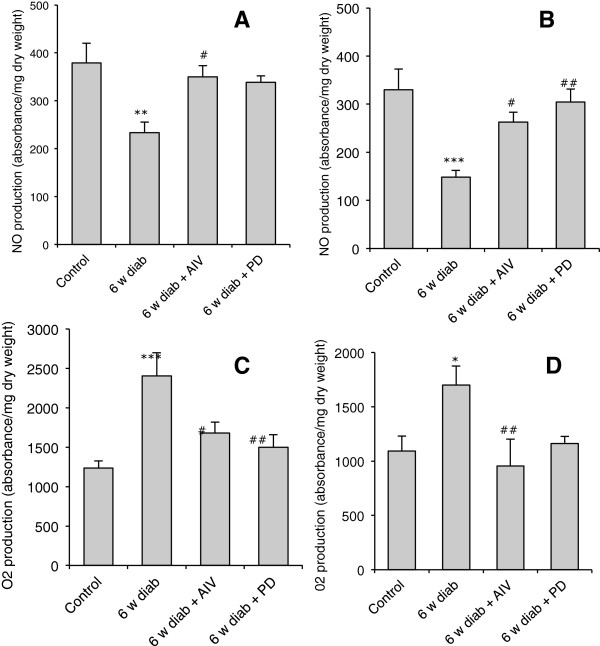
**Nitric oxide and anion superoxide production. (A**, **B)** Nitric oxide and **(C**, **D)** O_2_^-^ production in thoracic aorta sections (A, C) and mesenteric artery (B, D) of control mice, 6 weeks diabetic mice (6 w diab), and 6 weeks diabetic mice treated during the last two weeks with AngIV (6 w diab + AIV)) or PD 123319 (6 w diab + PD)). *p <0.05 and ***p < 0.001 *versus* control; # p < 0.05 and # # p < 0.01 *versus* 6w diab.

After 6 weeks streptozotocin-induced diabetes significantly decreased NO production both in the aortic (Figure 6A) and mesenteric (Figure 6B) vessels. In both vascular beds, treatment with AngIV or PD123319 prevented the decrease of NO induced by diabetes (Figure 6A and B).

The analysis of the production of superoxide anion showed that streptozotocin-induced diabetes significantly enhanced O_2_^•-^ production both in thoracic aorta (Figure 6C) and mesenteric arteries (Figure 6D), whereas AngIV or PD123319 prevented, respectively, the enhancement induced by diabetes in both vascular beds (Figure 6C and D).

### Effect of diabetes on EDR and media thickness in mice lacking the AT2 receptor

Treatment with streptozotocin induced a three-fold increase in glucose blood levels, associated with a significant decrease of body weight and systolic blood pressure of comparable amplitude in control wild type (Agtr2^+/+^) and AT2 receptor deleted (Agtr2^-/-^) mice (Table 1).

**Figure 7:**
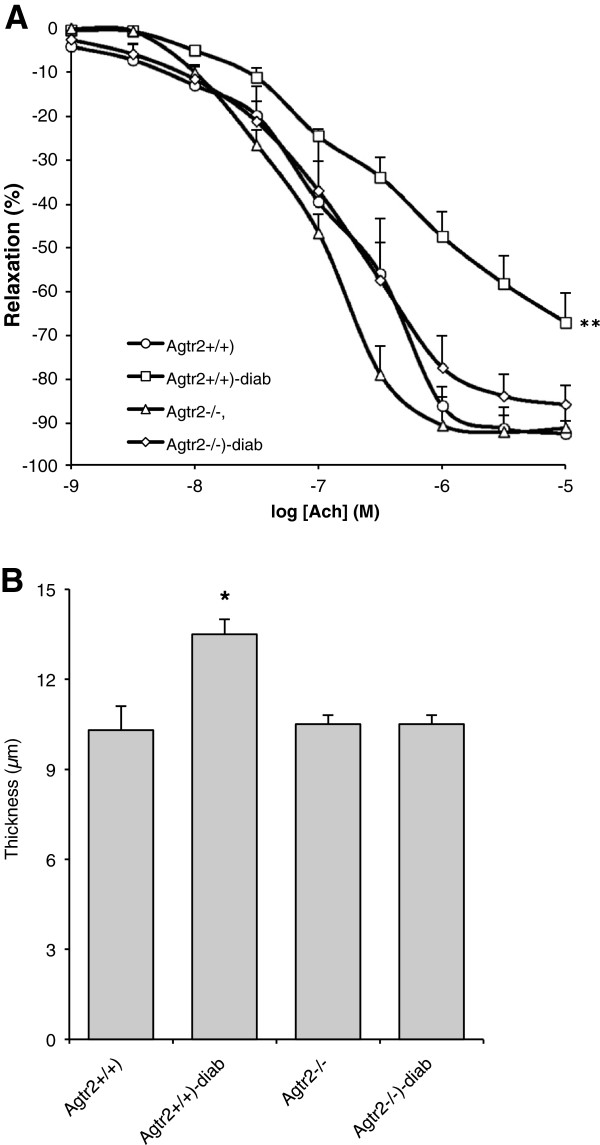
**Effect of diabetes in AT2R deficient mice.** Effect of 6 weeks diabetes on mesenteric arteries endothelial dependent relaxation **(A)** and media thickness **(B)** in wild-type (Agtr2^+/+^) and AT2R deficient mice (Agtr2^-/-^), treated (Diab) or not with STZ, n = 5 in each group; *p <0.05 and **p < 0.01 *versus* control.

Acetylcholine cumulative concentration–response curves were obtained after phenylephrine-induced preconstriction of mesenteric arteries of control wild type (Agtr2^+/+^) and AT2 receptor deleted (Agtr2^-/-^). While endothelium-dependent relaxation was markedly reduced by diabetes in Agtr2^+/+^ mice, no difference between control and diabetic group has been shown in Agtr2^-/-^ mice. In Agtr2^+/+^ mice, diabetes induced a significant increase of the mesenteric artery media thickness whereas no change has been shown in Agtr2^-/-^ mice between control and diabetic groups (Figure 7B).

## Discussion

The present study provides evidence that AT2 and AT4 receptors have opposite effects on vascular alteration caused by streptozotocin-induced diabetes in mice, a finding that provides new light on the complex role of the renin-angiotensin- system on the diabetes-induced vascular alteration, and may provide new therapeutic tracks for optimizing vascular prevention in diabetes patients.

Diabetes is associated with both microvascular and macrovascular diseases, manifested as altered vascular morphology and function. Thus, vascular smooth muscle cells (VSMCs) exposed to elevated glucose [23] and aortic smooth muscle cells from diabetic rabbits or rats exhibit increased *in vitro* growth rates [24]. Endothelial dysfunction is an early feature of diabetic vascular disease, characterized by a decrease in NO bioavailability and a concomitant increase in vascular O_2_^•-^ formation [25]. Loss of NO bioavailability precedes the development of overt atherosclerosis and is an independent predictor of adverse cardiovascular events [26,27].

In our study, induction of diabetes with streptozotocin in young mice induced the well-recognized vascular alterations, with a progressive dysfunction of endothelium-dependent relaxation and hypertrophy of the media in both aortic and mesenteric vascular beds, peaking after 6 weeks of diabetes duration. In this classical experimental model, we demonstrate that treatment with AngIV prevented the functional endothelial alteration and the vascular hypertrophy induced by diabetes. Moreover, when implemented in mice with already established vascular alteration, treatment with AngIV dose-dependently restored functional and morphologic vascular integrity. AngIV did not mediate its favorable actions *via* effects on metabolic or hemodynamic pathways such as glucose or blood pressure, respectively, two major pathways in the pathogenesis of diabetic vascular disease [28,29]. Consistent with the observed antitrophic effect, direct measurement by electron paramagnetic resonance showed that AngIV restored a normal balance between the content of NO and superoxide anion in the aortic and mesenteric walls. Taken together, our results fully confirm, and extend to the model of experimental type 1 diabetes the beneficial effect of chronic AngIV treatment first reported by Vinh *et al.* in Apo-E deficient mice [19], and further establish that AngIV acts by increasing NO bioavailability and decreasing oxidative stress.

However, our findings yield opposite conclusions as regards the respective contribution of AT2 and AT4 receptors in mediating the actions of AngIV. In the Apo-E deficient mice model, the protective effect of AngIV on endothelial dysfunction was attenuated by both the specific AT4 antagonist Divalinal and the AT2 antagonist PD123319, suggesting involvement of both receptors [18]. In a previous work, our group has reported that AngIV was protective in a model of acute ischemic stroke, and that both AT2 and AT4 antagonists likewise inhibited this protective effect [30]. In contrast in our diabetic mice, Divalinal completely blunted the protective effect of AngIV on EDR, whereas PD123319 had no effect. To further examine the role of the AT2 receptor, we thus studied the effect of AT2 pharmacological blockade in the absence of AngIV, and found that PD123319 had no effect on EDR in control mice, but completely blunted the diabetes-induced alteration of EDR. In the ApoE-deficient mice, AngIV blunted the increase of dihydroethidium staining for superoxide and PD123319 significantly inhibited this effect of AngIV. In sharp contrast we found that both AngIV and PD123319 equally inhibited diabetes-induced increase of superoxide production. Thus, the protective effect of AngIV in the atherogenic model of ApoE-deficient mice appears to be mediated by both AT2 and AT4 receptors, whereas in streptozotocin-induced diabetes mice, the protective effect of AngIV was solely mediated by AT4, while AT2 receptor stimulation seemed to play a detrimental role. This puzzling observation led us to extend our study and to further examine the role of AT2 by studying the effect of diabetes on vascular alterations in genetically modified mice lacking the AT2 receptor. In full consistence with the results gained with the pharmacological blockade of the AT2 receptor, we found that AT2 null mice were fully protected against the endothelial dysfunction and the vascular hypertrophy of the mesenteric arteries induced by six weeks of diabetes. In a study performed in ApoE/ AT1A receptor double knockout mice [31], chronic AT2 receptor inhibition with PD123319 increased plaque development, whereas direct AT2 receptor stimulation reduced atherogenesis, demonstrating, in accordance with Vinh et al., an antiatherosclerotic role of the AT2 receptor. However, Koitka *et al.*[32] reported that in ApoE-deficient mice, induction of diabetes with streptozotocin increased the aortic expression of the gene Agt2r, and was associated with a six-fold increase in plaque area that was significantly attenuated by both AT2R pharmacological blockade and AT2R deletion.

These patent discrepancies regarding the role of the AT2 receptor, that appears to be protective in the ApoE-deficient model of accelerated atherosclerosis, but detrimental in the setting of diabetes in fact no really surprising, and adds to a long list of controversial data. Left ventricular hypertrophy (LVH) is a major predictor of cardiovascular morbidity and mortality, and it is unanimously accepted that the angiotensin AT1 receptor is involved in the pathogenesis of hypertension and LVH, but the role of the AT2 receptor in LVH is still controversial. Studies addressing the involvement of the AT2 receptor in LVH performed in genetically altered, either AT2 receptor-deficient or AT2 receptor-overexpressing yielded highly controversial results with an equal number of studies supporting prohypertrophic, antihypertrophic, or neutral effects of the AT2 receptor in LVH [33].

These discrepancies have been discussed in deep elsewhere [34], and support the view that the mysterious versatility of the AT2 receptor phenotype appears to be highly cell-type and tissue specific, but also to depend on the autocrine/paracrine regulation of the cellular milieu of the target organs, and the specific experimental or pathophysiological conditions that determine the complex cross-talk between AT1 and AT2 receptors. For instance, You *et al. *[35] have shown that high blood pressure reversed the classical AT2 receptor-mediated vasodilation into vasoconstriction in spontaneously hypertensive rats, in agreement with a previous study showing that in young hypertensive rats AngII-induced contraction was decreased by AT2R blockade [36]. The mechanism of this reversal remains to be discovered, but may involve a switch in signaling from constrictor to dilator mechanisms due to increased endothelial AT2R expression. AT2R-dependent contraction in SHR is not affected by endothelium removal whereas AT2R-dependent dilation is abolished in the absence of endothelium. Thus the difference in the type of response might reflect a change in AT2R expression between the endothelium and the smooth muscle.

A recent study tested the effects of a 2 weeks treatment with the AT2 receptor agonist CGP-42112A on inflammation and oxidative stress in obese Zucker rats and compared them to their lean counterparts: the results suggested anti-inflammatory and antioxidative functions of AT2 receptor in obese Zucker rats, but proinflammatory and prooxidative functions in lean Zucker rats [37] further suggesting that AT2R function depends on the pathophysiological context.

In our study, diabetes was induced by streptozotocin, a convenient and widely used experimental model that is not devoid of criticisms and limitations. Noticeably, streptozotocin induces diabetes, but also elicits a strong and sustained inflammatory state. It is thus clearly possible that the deleterious effect of AT2R herein reported is the consequence of streptozotocin-induced side effects rather than that of diabetes per se, and the potential role of AT2R needs to be further studied in other experimental models of diabetes. Nonetheless, the observation that, in given experimental conditions, the AT2 receptor stimulation has a deleterious effect on vascular functional and morphological integrity challenge the concept that AT2 receptor stimulation represents the counter-regulatory arm of the RAS that unequivocally balances and opposes the effects of AT1 stimulation. Indeed, in spite of the uncertainties and discrepancies regarding AT2 functions, the view has emerged that the physiological functions of AT2 receptor is to antagonize the effects of the AT1 receptor [38], and that novel strategies to improve cardiovascular diseases may rely on drugs activating the tissue-protective arms of RAAS [39,40] The discovery of a first non-peptide, orally active AT2-receptor agonist compound 21 (C21), that should enter soon the first steps of clinical studies will help to clarify the therapeutic potential of AT2 stimulation [41], but our findings stress the need to cautiously delineate the specific pathological conditions in which it may prove beneficial, or in contrary may reveal potentially harmful.

During the last decade, AT4 has been identified as insulin-regulated aminopeptidase (IRAP) [42]. The nature of the molecular mechanism by which this membrane-bound enzyme mediates the variety of intracellular signaling and the biological effects triggered by AngIV remains so far obscure, and several hypotheses are still debated (see [43] for review). Interest on AngIV/AT4 (IRAP) pathway has nonetheless been boosted by its potential ability to enhance cognitive function and memory [44]. This potential interest as prompted active ongoing research to identify small biologically active non-peptides molecules that, like AngIV, inhibit the catalytic domain of IRAP [45]. Such new AT4 agonists will pave the way for innovative research for the prevention of cognitive decline, but also for stroke therapy [30,46,47]. Clearly our present findings, confirming the vascular protective effect of AngIV in type 1 diabetes mice also invite to consider AT4/IRAP as another potential target for revisited therapeutic strategies of RAAS modulation for cardiovascular disease prevention.

## Competing interests

The author(s) declare that they have no competing interests.

## Authors’ contributions

MN, NC and JJ executed the experiments, interpreted data, performed the statistical analysis and built the figures. LB administered the animal treatments and contributed to the experiments. SF performed the histomorphometric analysis and the electron paramagnetic resonance measurements and participated to the manuscript writing. JMA conceived and designed the study, and wrote the manuscript together with NO. All authors read and approved the final manuscript.
